# Second Birth Fertility in Germany: Social Class, Gender, and the Role of Economic Uncertainty

**DOI:** 10.1007/s10680-023-09656-5

**Published:** 2023-03-02

**Authors:** Michaela Kreyenfeld, Dirk Konietzka, Philippe Lambert, Vincent Jerald Ramos

**Affiliations:** 1 Hertie School, Berlin, Germany; 2 TU Braunschweig, Braunschweig, Germany; 3 Université de Liège, Liège, Belgium; 4 Université Catholique de Louvain, Louvain-la-Neuve, Belgium; 5 Humboldt University Berlin, Berlin, Germany

**Keywords:** Fertility, Germany, Uncertainty, Social class, Employment

## Abstract

Building on a thick strand of the literature on the determinants of higher-order births, this study uses a gender and class perspective to analyse second birth progression rates in Germany. Using data from the German Socio-Economic Panel from 1990 to 2020, individuals are classified based on their occupation into: upper service, lower service, skilled manual/higher-grade routine nonmanual, and semi-/unskilled manual/lower-grade routine nonmanual classes. Results highlight the “economic advantage” of men and women in service classes who experience strongly elevated second birth rates. Finally, we demonstrate that upward career mobility post-first birth is associated with higher second birth rates, particularly among men.

## Introduction

Classical demography has devoted substantial attention to the issue of class differences in marriage and fertility behaviour. Malthus (1998 [1798]) is unquestionably the most foundational scholar in this context. A general premise of his work is that there is a strong negative class–fertility gradient. He argued that the higher social classes, which at that time were composed of landlords and members of the aristocracy, would limit their number of children out of a “fear of lowering their condition in life” (ibid.: 6). He also assumed that although the lower social classes often lacked the necessary wealth and economic security to support a large family, their unrestrained sexual behaviour would result in high fertility. Malthus’ writings certainly reflect a striking degree of presumptuousness and a strong bias towards believing that the behaviour of his own social class was rational and conscientious (Petersen, 1990; Pullen, 2019). Nevertheless, his framework generated clear and testable hypotheses regarding the association between class, economic security, and fertility behaviour. Notestein (1936: p. 29) later elaborated on this perspective by asserting that class differences would “narrow or perhaps even reverse” if the fertility of the lower classes could be brought “more completely under control”.

In contrast to early classical demographic research (Brentano, 1910; Malthus, 1998 [1798]; Sallume & Notestein, 1932; Notestein, 1936), contemporary demography has devoted relatively little attention to the role of social class differences in fertility behaviour. Fertility researchers only rarely refer to this concept and instead tend to focus on differences in birth dynamics by education (e.g. Bartus et al., 2013; Nitsche et al., 2018; Nisén et al., 2021), earnings (e.g. Andersson et al., 2014; Heckman & Walker, 1990), or employment (Hofman et al., 2017; Matysiak & Vignoli, 2008). Further, the role of economic uncertainty in fertility has garnered substantial attention among fertility researchers (Vignoli et al., 2020), particularly in the context of the global financial crisis (e.g. Goldstein et al., 2013; Schneider, 2015, 2017) and the recent COVID-19 pandemic (e.g. Guetto et al., 2021b).

Given the increase in levels of labour market uncertainty, analyses of differences in behaviour across social classes may help to shed new light on contemporary fertility behaviour. Following the Weberian distinction between “class” (*Klasse*) and “status” (*Stand*), one’s “status” is rooted in social recognition and esteem (prestige). Social class is defined through people’s labour market positions, which are at the root of their long-term life chances, their economic vulnerabilities, and their employment risks (Erikson & Goldthorpe, 1992; Goldthorpe, 2007, 2010; Grusky & Sørensen, 1998). Within this framework, social classes represent firmly theorised and validated occupation-based categories that reflect economic uncertainties.

The main goal of this paper is to elaborate on the concept of social class in the analysis of contemporary fertility behaviour. Moreover, we provide empirical evidence on the relationship between social class and second birth rates in post-reunification Germany. Studying a single parity may be criticised as a “piecemeal approach” (Heckman & Walker, 1990, p. 1416). However, there are benefits related to such a “piecemeal approach” when the interest is in the relation between social class and fertility. Assuming that people typically achieve a certain class position by the time they have their first child, the advantage of focusing on the second birth is that it enables us to examine how people’s class mobility after the first birth affects their subsequent fertility behaviour. The analysis relies on a proportional hazard model in which we use a piecewise constant specification for the underlying process. Some concerns have been raised that proportional hazard models may conflate timing and quantum effects (Bartus et al., 2013; Kreyenfeld, 2002). Thus, we address these using a cure fraction model that disentangles the two components.

## Theoretical Considerations and Prior Research

### Prior Research on Uncertainty and Fertility

The Great Recession of 2008 has led to renewed scholarly interest in the role of economic uncertainty in fertility behaviour. This broad strand of the literature relies on objective measures of macroeconomic and labour market conditions, such as unemployment and fixed-term employment, as proxies for uncertainty (Alderotti et al., 2021). Recognising that uncertainty is high when economic conditions are dire, these studies generally agree that fertility rates are procyclical: i.e. they decrease during business cycle troughs, and increase during peaks (Adsera, 2005, 2011; Cazzola et al., 2016; Currie et al., 2014; Goldstein et al., 2013; Gozgor et al., 2021; Karaman Örsal & Goldstein, 2018; Sobotka et al., 2011). On the one hand, these short-run declines in period fertility may eventually translate into a “true” decline in completed cohort fertility, which implies a decrease in the total number of children that women of a certain cohort will have. On the other hand, these short-run declines in period fertility may be explained in part by postponement. For instance, Adsera (2011) found that first and second births occurred later in European countries that experienced high and persistent unemployment in the 1980s. However, one challenge that most of these studies encounter is that isolating fertility postponement (tempo effect) from a permanent decline in fertility (quantum effect) can be difficult (Sobotka et al., 2011).

In addition to unemployment rates, an important strand of the literature has also considered other measures of economic uncertainty, including GDP (Luci-Greulich & Thévenon, 2014; Matysiak et al., 2021), consumer confidence (Comolli, 2017; Schneider, 2015), and press coverage of economic developments (Gozgor et al., 2021; Guetto et al., 2021a; Schneider, 2015). These studies have also provided support for the claim that adverse economic conditions are negatively correlated with fertility. Particularly during the global financial crisis of 2007–08, which was characterised by sudden and steep increases in unemployment, firm closures, and, more broadly, negative reports on the state of the economy, Schneider (2015) found that states in the USA that were hit hardest by the recession also had the largest decreases in general fertility rates. The fertility declines in these states at the height of the recession were attributed not just to the overall increase in uncertainty and economic hardship in these areas, but also to the increase in contraceptive use among selected population subgroups, particularly among unmarried women and women from lower-income backgrounds (Schneider, 2015, 2017).

The findings mentioned above are complemented by an equally thick strand of the literature that has used subjective measures of economic uncertainty as determinants of fertility behaviour. These indicators are usually constructed from items in individual and household surveys that ask respondents whether they are worried about their own finances or the general state of the economy. Studies that used subjective measures of economic uncertainty have found that its effect on fertility is more nuanced; that is, that economic uncertainty seems to affect only select population subgroups. Kreyenfeld (2010) and Hofmann and Hohmeyer (2013) have both reported that there is little support for the claim that financial worries translate into first birth postponement. However, studies that have taken levels of education into account have shown that economic uncertainty accelerates the transition to the first birth among less educated women (Kreyenfeld, 2010, 2015). Indeed, there is strong evidence of differences in fertility behaviour in response to economic uncertainty by gender, population subgroup, and birth order.

In addition, a large body of research has examined how financial worries affect not just fertility *behaviour*, but fertility *intentions*. It has, for example, been shown that subjective economic uncertainty negatively affects birth intentions and that this relationship is more pronounced among men, given that men are often expected to take on a “primary provider role” (Busetta et al., 2019; Fahlén & Oláh, 2018; Kuhnt et al., 2021). Finally, a relatively recent body of research has also pointed to the role of future narratives of uncertainty as a determinant of fertility intentions (Brauner-Otto & Geist, 2018; Gatta et al., 2021; Vignoli et al., 2020).

### Social Class Position and Economic Uncertainty 

Many of the above-mentioned studies have grappled with the question of how a valid operational definition of economic uncertainty can be found. Having children is a long-term and binding commitment. Thus, it is not only people’s current economic conditions, but also their future employment prospects that influence their decisions about whether and, if so, when to have children. In this context, the concept “social class”, which is well established in research on social stratification and mobility, provides a potentially useful link. The theoretical backbone of contemporary class concepts is that in capitalist societies, individual life chances are essentially shaped by labour market, occupational, and employment conditions. Thus, social class is not interchangeable with education or income. Instead, it is a well-defined and “parsimonious indicator of the social positions of individuals” that helps us to “better understand fundamental forms of social relations and inequalities to which income is merely epiphenomenal” (Conelly et al., 2016: p. 3). Class researchers typically aggregate similar occupations into broader socio-economic class categories (Erikson et al., 1979; Goldthorpe, 2007; Oesch, 2006; Wright, 1985). Although class concepts differ with respect to their theoretical underpinnings and the basic mechanisms that are assumed to define and to distinguish classes, the prevalent class schemes, as developed by Erikson et al. (1979), Goldthorpe (2007), Wright (1985), and Oesch (2006), are aligned in terms of their basic occupational distinctions. For this study, the class schema proposed by Goldthorpe (2007) is particularly useful, as it suggests that occupational classes are inherently defined through employment relations. Accordingly, it is assumed that members of the same social classes have similar overall life chances, and are also exposed to similar degrees of economic vulnerability and uncertainty.

Goldthorpe (2007: pp. 110–118) differentiated occupations based on whether the related *tasks are difficult to monitor*, and by whether the *human assets* required for the occupations are specific. At the one extreme are occupations in which the tasks are difficult to monitor. People in these occupations usually have highly specific human assets (knowledge and expertise). At the other extreme are occupations in which the tasks are easy to supervise, and the quantity of work output is easy to measure. Furthermore, the human assets needed in these occupations are not specific. According to Goldthorpe (2007), the “nature of the tasks” and the “specificity of the human assets” determine the employment relationship. Based on this premise, he distinguishes nine categories, as described below (for details, see also the “Data, variables, and analytical strategy” section).^1^

*Unskilled and semi-skilled manual workers* [VIIa] and *lower-grade routine nonmanual employees* [IIIb] are often employed under short-term contracts. This implies that the jobs these categories take generally do not involve a long-term commitment from either the employer or the employee (Erikson & Goldthorpe, 1992: p. 41). Thus, the workers in these classes are subject to considerable economic uncertainty. The typical occupations in these classes include waiter, cleaner, shop assistant, housekeeper, taxi driver, and truck or van driver. In contrast to them, occupations in the *upper and lower service classes* [I, II] are mostly embedded in larger organisations, and “involve a longer term and generally more diffuse exchange” (ibid.: p. 103). Most importantly, the rewards associated with these occupations typically include “prospective elements”, such as employment security and “well-defined career opportunities” (ibid.: p. 103). Although the service classes have also been affected by the rise of fixed-term contracts, members of these classes generally enjoy greater employment stability than unskilled and semi-skilled labourers or routine nonmanual employees. These occupations include lawyer, scientist, engineer, higher-grade manager, in the German case also secondary school teacher (upper service class), as well as nurse, kindergarten teacher, technician, and lower-grade manager (lower service class). The *skilled manual workers* and supervisors [V, VI] as well as *the higher routine nonmanual employees* [IIIa] hold an intermediate position. These occupations involve mixed forms of employment relationships. The type of work done is either more difficult to monitor than un-/semi-skilled work, or it requires medium levels of specific human assets/human capital. The typical occupations in this category include machine operator, plumber, and electrician. Small employers/self-employed, farmers, and agricultural workers occupy separate classes [IVabc, VIIb]. These categories will not be considered in this investigation as they are small and heterogeneous. Although class concepts do not necessarily entail a hierarchical ordering (Conelly et al., 2016), social classes can be ranked by their degree of employment risk, with levels of economic vulnerability and uncertainty being highest among the un- and semi-skilled workers and lower-grade routine nonmanual employees, and lowest among the upper service class.

### Prior Research on Occupational Class and Fertility

While social class is a well-established concept in research on social inequality, only a relatively thin strand of recent literature has focused on the relationship between social class and fertility. Moreover, they have often adopted different strategies for classifying occupations. Some of the early studies, which were published when a large fraction of the population was still working in the agricultural sector, were particularly concerned with the elevated fertility of people working as farmers or farm labourers. An example is the study by Dinkel (1952), who argued that people in different occupations have different “ways of life” in terms of the practices and values that affect fertility. He showed that in the early twentieth century, farm owners and labourers had fertility rates that were 40–72% higher than those of professionals, depending on the region of residence in the USA. He attributed this gap in part to the labour needs of farming households (Dinkel, 1952; Maloney et al., 2014). Similar patterns have also been observed in Sweden in the mid-1900s, where farmers were shown to have the highest fertility rates among all occupational groups (Dribe and Scalone, 2014). More recent work has challenged these findings. For example, Köppen et al. (2017) found a drastic increase in childlessness among male farmers in France starting with the 1960s cohorts.

More recent research has also emphasised the importance of incorporating a gender perspective into explanations of relationships between social class and fertility (Szreter, 2015). It has been reported that since the 1990s in Sweden, women’s occupational class has had a U-shaped relationship with the transition to parenthood, with women in low-skilled and high-skilled occupations having higher birth risks than women in medium-skilled occupations (Dribe & Smith, 2021). Research on Austria has found that women whose educational levels typically lead them to have lower-class occupations are less likely to remain childless than women whose educational levels lead them to have higher-class occupations (Neyer et al., 2017). Begal and Mills (2013) used data from the Netherlands to study the birth behaviour of women of the 1940–1985 cohort by groups of occupations and found that women in teaching-related occupations transitioned relatively quickly to first birth. Their results also indicated that women in communicative jobs (healthcare, teaching) transitioned relatively rapidly to higher-order fertility, while women in technology-related occupations had comparatively low higher-order birth risks.^2^

The studies that come closest to using the established sociological concepts of social class and analysing its relation to fertility generally find a positive relationship of being in a higher social class with second-order births (Baizan, 2020, 2021; Ekert-Jaffe et al., 2002). Using event history and simultaneous equation models on longitudinal data reveal elevated risks of women in the higher professional class in the transition to second birth in Spain (Baizan, 2020), as well as Austria, France, Norway, and the UK (Baizan, 2021). Gender also matters in this nexus—Ekert-Jaffe et al. (2002) find no strong variation in women’s fertility depending on their social class. However, they observed that the second birth rates of women with a spouse in a higher managerial position were well above average. Building on this strand of the literature, we likewise incorporate in our analysis the role of gender and economic uncertainty.

### Hypotheses

As Goldthorpe (2007) argued, social classes are based on people’s occupations and employment positions, which provide them with differing levels of socio-economic resources, including with varying degrees of employment security. Employment security is rooted in the nature of the job-related tasks the employee is expected to perform and in the kind of job contract deemed necessary to incentivise the employee to perform the tasks. Accordingly, class positions differ with respect to employee–employer commitment levels and trust relationships, and in terms of the long-term character of employment contracts. Assuming that fertility choices are long-term, binding biographical decisions that require some degree of economic certainty, it can also be assumed that fertility behaviour differs by social class. Given the more advantaged positions of the upper service class, individuals in this class should have the highest second birth rates, while the semi-/unskilled workers and the lower-grade routine nonmanual employees should have the lowest second birth rates (*hypothesis 1*).

Compared to their income and earnings, peoples’ class positions are rather stable traits that mirror their long-term employment and lifetime chances. However, the childbearing years coincide with a period in people’s lives in which they are typically seeking to advance in their professional career or are participating in education or vocational training. In Germany, as in most other European countries, the age at first birth has risen to about age 30 for women and to about age 32 for men. Although the scholarly literature often assumes that class positions are rather fixed beyond age 30, upward mobility—and, to a lesser extent, downward mobility—may occur beyond that age. As having a higher-class position is linked to greater economic security, we assume that upward mobility will lead to higher second birth rates (*hypothesis 2*).

Traditional concepts have understood social class as a household concept, where the social class of an individual in a household is defined over the social class of the (male) prime earner (Goldthorpe, 1983). Feminist scholars have challenged that view, calling for a gender and individual perspective on social class (see, for example, Baxter, 1994; Bonney, 2007: p. 146). We define social class as an individual trait defined over one’s own occupation. Still, labour market options are strongly gendered in most societies, also in the case of reunited Germany, which is the focus of this investigation. Important family policy reforms were enacted in recent years in Germany, most notably the expansion of childcare in 2005 and the reform of parental leave benefits in 2007. Scholars have argued that these reforms have been consequential, as they represent a sharp departure from Germany’s previously well-established path of providing policy support for a conservative family model centred on the male breadwinner (Fleckenstein, 2011). While the full-time employment rates of mothers have increased in recent years, employment patterns after the first birth are still strongly gendered, particularly in West Germany. Against this background, we assume that social class is a stronger predictor of men’s than of women’s fertility transitions (*hypothesis 3*).

Finally, people’s class positions reflect their long-term employment chances and their levels of economic security and vulnerability. Among the benefits of the German Socio-Economic Panel (GSOEP) dataset, which we will use in our investigation, is that it includes not only measures of social class, but also items that estimate levels of economic uncertainty and vulnerability, such as the subjective feeling of having financial worries. This information allows us to study whether and, if so, how this measure correlates with the respondents’ social class positions. It also enables us to explore whether the effect of social class is robust to the inclusion of more direct measures of uncertainty. Generally, we expect to find that having financial worries may explain some of the class differences. Thus, we assume that the effect of social class becomes weaker after controlling for other measures of uncertainty (*hypothesis 4*).

Beyond these four guiding research hypotheses, the analyses will take into account the possibility that social class has a distinct influence on *the timing and the quantum* of second birth fertility. Because they tend to be older when they have their first child, members of the service class are likely to face a “time squeeze” that leads them to progress more rapidly to the second birth than, for example, members of the unskilled and semi-skilled and the routine nonmanual classes, who often have their first child at a younger age. We will use a cure fraction model to check whether a more fine-grained modelling approach that differentiates between timing and quantum effects generates the same results as standard event history models.

## Data, Variables, and Analytical Strategy

### Data and Analytical Sample

Data for this investigation come from the German Socio-Economic Panel (GSOEP) release 37 (Socio-Economic Panel, 2022). The GSOEP is a yearly household panel that was launched in 1984. The original sample includes West German respondents and an oversample of migrants from the former labour recruitment countries. Since its inception, various subsamples have been added to this dataset, most prominently an East German subsample in 1990. For this investigation, we use data from the years 1990 to 2020.^3^ Thus, the investigation covers post-reunification Germany. We limited the sample to respondents who had a first child from 1990 onwards, and provided valid information on each individual’s social class in the year of the first birth. We omitted respondents who were self-employed or farmers when they had their first child, as this group was rather small and heterogeneous. Finally, we censored the cases 12 years after the first birth, and restricted the sample to episodes in which the respondents were aged 18–55. The final analytical sample includes 2282 men who fathered 1161 children, and 2819 women who contributed 1371 births to the study population.

### Variables

#### Dependent Variable

The dependent variable is the transition to the second child, with the process time being measured in months from the birth of the first child to the start of the second pregnancy (i.e. the date of childbirth backdated by nine months). In some cases, the month of childbirth was missing from the data. We imputed the missing information using a random number generator. While we had information on the month of childbirth, information on the time-varying covariates (such as financial worries and subsequent class positions) was available only at the time of the interview. We assumed in these cases that the respondents’ characteristics were fixed until the next interview. Figure 2 in the appendix plots the survival curves to the second child by social class and gender. The figure suggests that when the first child was age 10, the probability of having a second child was around 63% which is a bit lower than recent official estimates (Statistisches Bundesamt, 2019).^4^

#### Social Class

The key variable of interest is the *class position in the year of first childbirth,* which we treat as a time-constant covariate in the investigation. We have chosen to focus on the class position at first birth in part because it allows us to examine how downward or upward mobility relates to fertility behaviour (see below). We have operationalised the class position using the Erikson Goldthorpe Portocarero (EGP) class schema which has been adopted in the GSOEP (Erikson et al., 1979). We have removed from the sample the self-employed and agricultural labourers because they were a very heterogeneous group, and because they comprised only a very small fraction of the population. We also grouped routine service workers and unskilled manual workers into a single category because the shares of men employed in routine service jobs and the shares of women employed in unskilled manual jobs were extremely low. If a person was not employed in the year when s/he had a child, we used the person’s class position in the previous year. However, some respondents, particularly women, were not working both in the year when they had a child and in the previous year. For these cases, we built a separate category that indicates that the person was not employed (including a small fraction of individuals who were in education). In total, we distinguished the following five class positions:

- Upper service class [I],
- Lower service class [II],
- Skilled (manual and higher-grade routine nonmanual) classes [IIIb, V, VI],
- Semi- and unskilled (manual and lower-grade routine nonmanual) classes [IIIa, VIIa],
- Not employed (including individuals in education).

Among men, the largest group is composed of persons who were in the skilled (manual and higher routine nonmanual) class, while the lower service class is the most common class for women at first birth (see Table 5 in Appendix). A larger fraction of men than of women were in the upper service class at the time of their first birth. For both women and men, typical occupations in the upper service class are medical doctors or public service administrative professionals. There is a higher fraction of computer systems designers and engineers among the men, while architects and sales, marketing and department managers are more prevalent among women in the upper service class. A striking difference between women and men furthermore is that more women than men were not employed in the year when their first child was born (or in the prior year).

#### Partner’s Social Class

We also account for the partner’s social class at first birth in some parts of the investigation. Unfortunately, there is a large fraction of the respondents for which we do not have any information on partner’s social class (see Table 5). For those respondents for which information is available, we observe a strong class homogamy on the couple level (see Tables 7 and 8). For example, 48% of the women who were employed in the upper service class around the time of first birth had partners who were also in the upper service class.

#### Social Mobility

To capture the effects of *social mobility,* we have generated a time-varying variable that combines the class position in the year of first childbirth and the class position in the years thereafter. We distinguish between individuals who remained in the position they had in the year of their first birth and individuals who experienced upward or downward mobility (compared to the class position in the year of first birth). Here, we assume the following hierarchy of class positions: upper service class > lower service class > skilled manual/higher-grade routine nonmanual classes > unskilled and semi-skilled manual/lower-grade routine nonmanual classes. A separate category includes the respondents who were not employed either at the first childbirth or in the period that followed. Thus, we do not consider the move from nonemployment at the first birth to participation in the labour market as upward mobility. Tables 1 and 2 report the sample statistics for social mobility by class and gender. Within the given class framework (disregarding nonemployment), it was not possible for members of the upper class to move up, or for members of the semi-skilled and unskilled classes to move down. Thus, upward mobility was possible for the lower and medium social classes only, while the semi-skilled and unskilled classes did not contribute any exposure time to the downward moves. These floor and ceiling effects need to be considered when interpreting the model results (see also Breen & Müller, 2020). The table provides some relevant insights into the class-specific mobility patterns. While upward moves were rare for women after first birth, a significant fraction of the men—particularly those who were previously in unskilled or semi-skilled occupations—experienced upward mobility after first birth.

**Table 1:** Financial worries by social class, distribution by person-years, men, column % (Source: SOEP, v37, 1990-2020, unweighted estimates)

	Men’s social class in the year of the first birth				
	Upper service	Lower service	Skilled	Semi-/unskilled	Not employed
*Social mobility*					
Stable	86	73	71	66	–
Upward	–	11	8	19	–
Downward	11	11	9	–	–
Not employed	3	5	11	14	100
*Financial worries*					
Very worried	9	13	24	27	34
Somewhat worried	50	51	54	55	49
Not worried	41	36	22	18	17
*Age at first birth*					
Mean	34.1	33.5	29.9	29.3	27.8
N (person-months)	17,687	21,142	40,722	22,389	6,301

**Table 2:** Financial worries by social class, distribution by person-years, women, column % (*Source*: SOEP, v37, 1990–2020, unweighted estimates)

	Women’s social class in the year of the first birth				
	Upper service	Lower service	Skilled	Semi-/unskilled	Not employed
*Social mobility*					
Stable	52	49	42	40	–
Upward	-	3	6	10	–
Downward	13	10	6	–	–
Not employed	35	38	45	50	100
*Financial worries*					
Very worried	11	16	22	30	29
Somewhat worried	54	57	60	55	52
Not worried	35	27	19	15	19
*Age at first birth*					
Mean	32.1	30.3	28.5	27.0	25.0
N (person-months)	11,292	34,791	32,451	33,721	20,093

Note: Sample includes persons at risk of a second birth. For the distribution of financial worries, person with missing information for that particular variable was excluded

#### Economic Uncertainty

The GSOEP includes various measures of economic uncertainty, which allows us to examine how these measures are correlated with class positions. In our analysis, we use the subjective feeling of having *financial worries.* The respondents’ financial worries were operationalised with a survey question that asked them whether they were very worried, somewhat worried, or not worried about their personal financial situation. In line with our theoretical expectations, we observe that the respondents’ financial worries varied considerably depending on their class position. For example, individuals who were in a semi-skilled or an unskilled position were three times more likely than members of the upper service class to report being very worried about their economic situation (Table 1 and 2). Moreover, individuals who were not working at the first birth also indicated that they were very concerned about their economic situation. The patterns were similar for both women and men, but the association was slightly stronger for the women than for the men (Spearman’s rank correlation coefficient was −0.23 for women and −0.17 for men).

#### Socio-Demographic Covariates

The regression analysis also controls for the standard socio-demographic variables. (The table with the complete summary statistics is included in the appendix; see Tables 5 and 6.) We account for *the duration since the previous birth* (baseline hazard). Region is included in the models by distinguishing between West and East Germany (including West Berlin). We also control for migration background and differentiate between natives and individuals with a *migration background*. Education was distinguished between low (lower than vocational degree), medium (vocational degree), and higher education (university degree). *Age at first childbearing* is another important predictor for subsequent births. This variable may be strongly correlated with social status, and it may have a distinct influence on the timing and the quantum of second birth fertility. Table 1 and 2 provide support for the assumption that there was a strong age gradient in first childbearing by social class. The average age at first childbearing was much higher among the respondents in the upper service class than among the respondents in the manual and routine occupations.

### Analytical Strategy

#### Main Analysis

Our analytical strategy consists of two steps. In a first step, we use a piecewise constant model to estimate the transition rates to the second child. The piecewise constant model is particularly suitable for studying vital events, such as birth dynamics (Hoem, 1987; Hoem & Hoem, 1989). Like the Cox model, it belongs to the large group of proportional hazard models. However, unlike the Cox model, it provides parameter estimates for the baseline hazard, which allows the researcher to better grasp the underlying process. The baseline hazard in our analysis is the duration since the last birth. The baseline *h*_*0*_(t) is partitioned into different pieces, while the hazard is assumed to be constant within the respective segments. In our model, time $t$ is partitioned into four segments: i.e. into 0–23, 24–47, 48–71, and 72–143 months after the first childbirth. The hazard at time $t$, given a set of $X$ covariates, is defined as:

1 $$h(t|X)={h}_{0}(t)\times \mathrm{exp}(\beta x)$$

while the baseline hazard is defined as follows:

2 $$h_{0} \left( t \right) = \left\{ {\begin{array}{*{20}l} {h_{1} ,t(\left. {0,\tau_{1} } \right],} \hfill \\ {\begin{array}{*{20}c} {h_{2} ,t(\left. {\tau_{1} ,\tau_{2} } \right],} \\ {h_{3} ,t(\left. {\tau_{2} ,\tau_{3} } \right],} \\ \end{array} } \hfill \\ {h_{4} ,t(\left. {\tau_{3} ,\tau_{4} } \right]} \hfill \\ \end{array} } \right.$$

The main covariates in the first model (M1 for men and W1 for women) are the variables for social class position and class mobility. In a second model (M2 for men and W2 for women), we also account for the subjective feeling of uncertainty. In a final model (M3 for men and W3 for women), we furthermore account for partner’s social class.

#### Robustness Checks

The descriptive statistics (see Tables 1 and 2) show that the age at first birth varied systematically by social class. This finding may have implications for the model’s assumptions. Individuals who were older when they had their first child may have faced a “time squeeze” that led them to progress rapidly to having a second child. Thus, the elevated birth rates may indicate that individuals who were older when they had their first child were less likely to have a second child, but spaced their first and second births closer together. As the age at first childbearing and social class are closely related, the failure to properly model the influence of the age at first birth on second birth rates may lead to biased results.

We employ a cure fraction model to separate the timing and the quantum effects. There are two broad families of cure fraction models. First, the mixture cure model (Berkson & Gage, 1952) that relies on a survival function written as a mixture of two components: one corresponding to the proportion of “immune” subjects in the population (those who never have a second child) and a second one corresponding to the survival function of the population who will experience the event (see, for example, Beaujouan & Solaz, 2013 in a fertility context). Second, the promotion time model, also named the bounded cumulative hazard model (Yakovlev and Tsodikov, 1996), which has recently been employed in fertility research as well (e.g. Bremhorst et al., 2016). This approach explicitly acknowledges that the population survival function $${S}_{p}\left(t\right)$$ converges (when $$t>T)$$ to a nonzero value $$\pi$$ corresponding to the “immune” fraction. In our context, $$T$$ would denote the minimum number of months after the first birth after which it is reasonable to conclude that subjects with one child will not have a second, $$\pi$$ denoting the expected proportion of subjects in this situation. It implies that the cumulative hazard function $${H}_{p}\left(t\right)$$ is bounded by $$\theta =-\mathrm{log}(\pi )$$ and can be written as $${H}_{p}\left(t\right)=\theta F\left(t\right)$$ where $$F\left(t\right)$$ is a cumulative distribution function such that $$F\left(0\right)=0$$ and $$F\left(t\right)=1.0$$ when $$t>T$$. The associated density function $$f\left(t\right)$$ can be viewed as a normalised form of the population hazard function $${h}_{p}\left(t\right)= \theta \times f(t)$$ governing the dynamics of events. A flexible spline-based form for $$f\left(t\right)$$ will be considered. Covariates $$x$$ can enter the specification of $$\theta$$ and $$F\left(t\right)$$ using $$\theta (x)=\mathrm{exp}({\beta }_{0}+{{\beta }^{^{\prime}}x)}$$ and $$S\left(t|x\right)=1-F\left(t|x\right)$$=$${S}_{0}{(t)}^{\mathrm{exp}({\gamma }^{^{\prime}}x)}$$.^5^ The promotion time model has been formalised for models with time-constant covariates, but the inclusion of time-varying covariates is still an emerging topic in the statistical literature (see Lambert and Bremhorst (2020) and Lambert and Kreyenfeld (2023) for recent proposals). For this reason, we have fixed the values found for education and region at the first birth. As this is not meaningful for social mobility, we omit this variable from this part of the investigation. We also omit financial worries. One could fix this variable at first birth, but the meaning of this variable as a fixed and time-varying covariate would differ.^6^

## Results

### Social Class, Social Mobility, and Second Birth Fertility

Table 3 reports the results from a set of event history models that estimated the transition rate to the second child. All models report a consistent pattern with respect to the control variables: i.e. the second birth rates are highest two to three years after the first childbirth. Furthermore, there is a strong negative correlation between the age at first childbirth and the progression rates to the next childbirth. Moreover, the second birth rates are roughly 40% lower in East than in West Germany. This finding is very much in line with prior research on the East–West differences in higher-order childbearing behaviour (Arránz Becker et al., 2010). We find no relevant differences between native and migrant populations. This may be surprising, as it is often assumed that the migrant population has higher fertility than the native population. It should be noted that this study focuses on second-order births, for which native migrant differences tend to be less pronounced. Furthermore, apart from migrants of Turkish origin, many of the more recent migrants in Germany come from Central and Eastern European countries that are characterised by low second birth rates.

**Table 3:** Piecewise constant event history model. Relative second birth risks (hazard ratios) Source: SOEP, v37, 1990–2020. Own unweighted estimates

	Men		Women	
	M1	M2	W1	W2
*Age of first child*				
Age first child 0–1	Ref.	Ref.	Ref.	Ref.
Age first child 2–3	1.66***	1.65***	1.58***	1.59***
Age first child 4–5	0.82**	0.81**	0.85	0.86
Age first child 6–11	0.32***	0.32***	0.34***	0.34***
*Age at first birth*				
Age 18–23	1.04	1.04	1.13	1.15
Age 24–28	1.15*	1.15*	1.04	1.05
Age 29–32	Ref.	Ref.	Ref.	Ref.
Age 33–55	0.71***	0.71***	0.48***	0.48***
*Region*				
West Germany	Ref.	Ref.	Ref.	Ref.
East Germany	0.60***	0.60***	0.66***	0.68***
*Migration background*				
Native	Ref.	Ref.	Ref.	Ref.
Migration background	1.02	1.03	1.09	1.10
*Social class at first birth*				
Upper service	Ref	Ref	Ref	Ref
Lower service	0.79**	0.79***	0.75***	0.76***
Skilled	0.78**	0.78***	0.81*	0.83*
Semi-/unskilled	0.58***	0.58***	0.62***	0.64***
Not employed	0.86	0.86	0.64***	0.64***
*Education*				
Low (no degree)	Ref.	Ref.	Ref.	Ref.
Medium (vocational)	1.09	1.08	1.22**	1.22**
High (university)	1.33**	1.34**	1.39***	1.37***
*Social mobility*				
Upward	1.26**	1.27**	1.32*	1.31
Stable	Ref.	Ref.	Ref.	Ref.
Downward	0.88	0.88	1.00	1.01
Not employed	0.89	0.90	1.17**	1.18**
*Financial worries*				
Very worried		1.02		0.80***
Somewhat worried		1.15**		0.87**
Not worried		Ref.		Ref.
*Sample size*				
Person-months	108,241	108,241	132,348	132,348
Events	1161	1161	1371	1371

Note: Further variables in the model for “other” social mobility and “missing” for financial worries and education. *p < 0.1; **p < 0.05; ***p < 0.01

The analysis shows that the men’s social class is strongly related to their second birth behaviour (Model M1): i.e. the lower the social class, the lower the second birth rate. The groups that stand out are the semi-skilled and unskilled classes, as their second birth rate is 42% lower than that of the reference group (upper service class positions). The pattern for women is similar, but the differences are attenuated (Model W1). Class mobility also plays out differently for women and men. Among men, upward mobility is associated with an increase of roughly 25% in the second birth rate, while downward mobility and nonemployment are unrelated to the second birth rate. The parameter for upward mobility for women is in the same direction and of similar magnitude as for men, but it is only weakly significantly different from the reference category (stable class position). This weak significance may not come as a surprise, given the small fraction of women who experienced social upward mobility after their first birth (see Table 6 in appendix).

Models M2 and W2 display the results from the models that include the additional measures of economic uncertainty (subjective financial worries). While the men’s financial worries do not seem to influence the second birth rates (Model M2), the women’s financial worries are associated with a postponement of second childbearing (Model W2). The second birth rates of women who reported being very worried are 20% lower than those of women who reported being not worried. Women who said they are somewhat worried have a birth rate that is 17% lower than those without worries. The effect size seems strong, and the inclusion of this variable also increased the model fit (log-likelihood ratio test was conducted, *p* < 0.05). However, it does not greatly affect the class pattern. Thus, among women, there seems to be an independent effect of subjective worries that is not captured by their own class position.^7^

### Partner’s Social Class and Second Birth Fertility

**Fig. 1:**
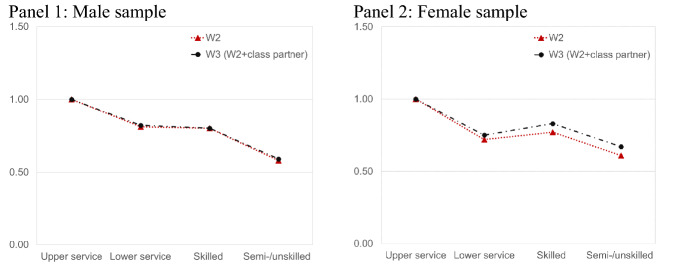
Piecewise constant event history model. Relative second birth risks (hazard ratios). Note: The analysis was conducted on the sample with respondents with valid information on partner’s class position (n = 81,669 person-months for male sample; n = 83,654 person-months for female sample). Further variables in the model are age of first child, age at first birth, education, region, migration status, own social class, social mobility, and financial worries. Hazard ratio for “not employed” is not displayed in the figures due to the small sample size in the male sample. *p < 0.1; **p < 0.05; ***p < 0.01. (*Source*: SOEP, v37, 1990–2020. Own unweighted estimates)

**Fig. 2:**
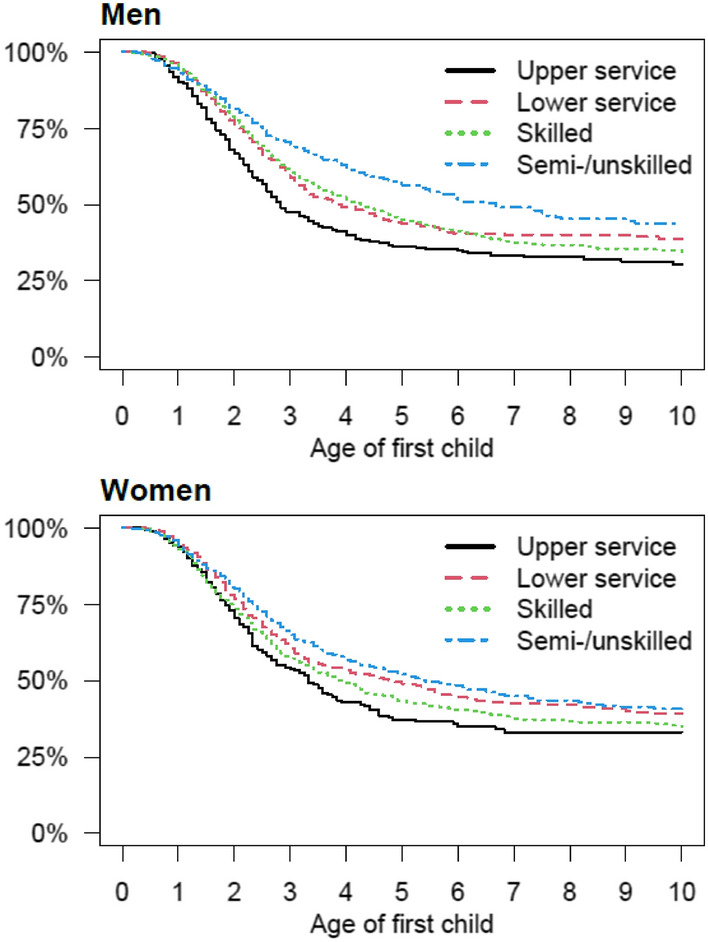
Kaplan–Meier survival functions to the second birth by social class at the first birth and gender. Note: It should be noted that the survivals are not weighted. As the GSOEP oversamples certain groups, such as migrant populations and East Germans, caution is advised when considering the descriptive results, as they do not control for migration status and region (as is done in the multiple regression). Individuals who were unemployed when they had their first child are not included, as they represent an only small fraction of the men, and including them would have resulted in unstable estimates of the survival curves. (*Source*: SOEP, v37, 1990–2020. Own unweighted estimates)

Figure 1 displays the results from the models which additionally controls also for the partner’s class position. The figures include the hazard ratios from the model that includes the partner’s class (M3 and W3) as well as results from a model that does not include it. It contains furthermore the same covariates as in the prior analysis (M2 and W2). Note, however, that this part of the analysis was restricted to respondents with valid information on partner’s class characteristics so that the parameters slightly deviate from the previous investigation. As can be depicted from the figure, patterns for the male sample remain unchanged regardless of whether partner’s social class is controlled or not. However, patterns are attenuated for the female sample, suggesting that men’s social class has a more positive impact on second birth transitions than women’s in the German context. Still, the overall pattern remains unchanged: The higher the social class of both women and men, the higher is the second birth rate.

### Timing and Quantum Effects

Table 4 reports the results from the cure fraction models. The model results for the male sample show that the previously reported “class effects” are mainly quantum effects. Thus, we can ascertain that there is a positive class and fertility nexus. The members of the upper service class are the most likely to progress to the second birth, and the semi/unskilled workers and lower-grade routine nonmanual employees are the least likely to have a second child. We also find that the age at first fatherhood has a distinct influence on the timing and the quantum of male second birth fertility. While a late age at first fatherhood leads to the first and second child being more closely spaced, it also lowers the quantum of fertility. We find that a migration background increases quantum, but the parameter is only significant  in the male sample. Further, the reduced hazard rate that we found for East Germany in the previous investigation seems to be related to both timing and quantum effects. Further, men’s higher levels of education seem to affect second birth quantum, but not timing.

**Table 4:** Cure fraction model. Relative second birth risks (hazard ratios). (*Source*: SOEP, v37, 1990–2020. Own unweighted estimates)

	Men		Women	
	Quantum	Timing	Quantum	Timing
*Age at first birth*				
Age 18–23	1.16	0.77	1.49***	0.60***
Age 24–28	1.24***	0.84*	1.19**	0.74***
Age 29–32	Ref.	Ref.	Ref.	Ref.
Age 33–55	0.61***	1.45***	0.44***	1.45***
*Region*				
West Germany	Ref.	Ref.	Ref.	Ref.
East Germany	0.62***	0.85	0.73***	0.77***
*Migration background*				
Native	Ref.	Ref.	Ref.	Ref.
Migration background	1.13*	0.82**	1.08	1.02
*Education*				
Low	Ref.	Ref.	Ref.	Ref.
Medium	1.10	1.18*	1.28***	0.98
High	1.38**	1.10	1.39***	1.06
*Social class at first birth*				
Upper service	Ref.	Ref.	Ref.	Ref.
Lower service	0.85*	0.86	0.72***	1.13
Skilled	0.90	0.78**	0.77**	1.27**
Semi-/unskilled	0.64***	0.90	0.61***	1.16
Not employed	0.78	1.12	0.61***	1.42**
Null model (no covariates)	− 6268		− 7446	
Final model	− 6190		− 7345	
Person-months	127,701		158,161	
Events	1171		1381	

Note: *p < 0.1; **p < 0.05; ***p < 0.01. Person-months and events are slightly different from Table 2 as the analysis was not censored after 12 years

In many respects, the results for the female sample concur with the results for the male sample. Most of the class differences can be attributed to quantum effects. A pronounced pattern is found for women who were not employed in the year of the first childbirth: i.e. they are rather unlikely to have a second child, but if they have a second child, they often have it at short durations after first birth. The age at first childbearing has the same effect in the female sample as in the male sample. An early age at childbearing increases the quantum, but it also increases the birth interval. Late childbearing has the opposite effect, as it lowers the quantum, but it shortens the birth interval.

## Conclusion

While classical demography had a strong interest in the relationship between social class and fertility, contemporary fertility research rarely uses the class concept to investigate birth behaviour. Instead, scholars mostly focus on income, education, and employment when examining how labour market conditions are related to fertility behaviour. However, the global financial crisis, the COVID-19 pandemic, and, more recently, the recession that is expected to follow the Russian war of aggression in Ukraine have led to increasing scholarly interest in the uncertainty and fertility nexus. Nonetheless, there is still considerable ambivalence about how to properly operationalise economic uncertainty and long-term employment chances. In this context, it is conspicuous that most demographers have failed to take into consideration the large body of sociological work on the relationship between social class, economic vulnerability, and life chances. We argued in this paper that social class is a well-theorised concept with firmly validated categories that has been effectively employed in sociological labour market research and that can also prove useful in demographic investigations.

The empirical part of this investigation focused on second birth fertility in post-reunification Germany (1990–2020). We chose to look at second childbearing in order to explore how class mobility after the first childbirth affected birth behaviour. The results of the descriptive investigation indicated that women were less likely than men to experience upward mobility. Furthermore, while we found that moving up the social ladder increased men’s second birth risks significantly, we only observed a statistically weak association between women’s mobility and their second birth fertility. We also found that the association between social class and second birth fertility was stronger in the male than in the female sample. Nevertheless, the overall pattern was similar for both genders, with members of the upper service classes having the highest birth rates and members of the unskilled manual/lower routine nonmanual classes having the lowest birth rates. We also examined the question of whether subjective feelings of uncertainty explained the differences by class. Our findings indicated that while having financial worries was associated with lower second birth rates, particularly among the female sample, the inclusion of this variable did not change the class patterns. An important methodological question we considered was whether the model results would be robust if timing and quantum effects were differentiated. To this end, we employed cure fraction models. The cure fraction model showed that the age at first childbearing had a very different impact on the timing and the quantum of fertility: i.e. a later age at childbirth reduced the quantum, but it led to a closer spacing of the first and the second child. The model also showed that the class differences were mostly quantum effects.

While our investigation generated novel results on the class–fertility nexus, there are important limitations to this investigation that should be mentioned. *First,* we focused on second births. As the transition to the first birth usually coincides with the phase of life when people are getting established in the labour market, first birth analyses would have required additional considerations. Moreover, as higher-order births are rare in Germany, we would not have sufficient case numbers to study third- or higher-order births. Thus, while our focus on second births may be justified, the analysis of  a single transition may still be characterised as a “piecemeal approach” (Heckman & Walker, 1990, p. 1416). We cannot rule out the possibility that the patterns for other birth parties are different from the patterns we found for second births. Furthermore, there may be selection into the study population based on social class characteristics. Earlier studies have revealed that highly educated women in German are more likely to remain childless than less educated. As social class and education are correlated, one may conclude that women who belong to the upper service class and are at risk of second birth are a selective population. It may be that it is their particular characteristics that have selected them into the pool of mothers also drive their higher progression to the next child (see, for example, Bartus et al., 2013; Kreyenfeld, 2002). *Second*, we assumed that social class is a solid and firmly validated indicator of economic uncertainty, economic vulnerability, and long-term life chances. The GSOEP offers various additional variables that indicate different facets of economic uncertainty and economic standing (e.g. labour market earnings, term-limited working contracts, worries about global economic development). Among the many variables that this dataset offers, we picked having financial worries to illustrate how social class correlates with other measures of economic insecurity. We included this variable in our model, but it did not ultimately explain much of the class differences. The “stepwise procedure” we used may be criticised for failing to sufficiently account for other measures of uncertainty that may affect the relationship between social class and fertility behaviour. Possibly, more elaborated mediation analysis that account for the complex interplay of social class and various dimensions of uncertainty may be a way forward here (Kuha et al., 2021).

## Figures and Tables

**Fig. 3:**
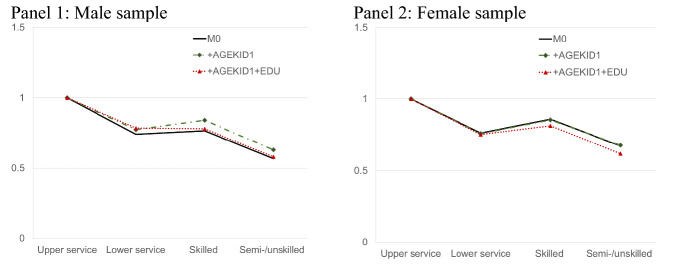
Robustness Checks: Piecewise constant event history model. Relative second birth risks (hazard ratios). Note: (n = 108,241person-months for male sample; n = 132,348 person-months for female sample). Hazard ratio for “not employed” is not displayed in the figures due to the small sample size in the male sample. M0: Further variables in the model are age of first child, region, migration status, social mobility. M0 + AGEKID1: M0 + age at first birth. M0 + AGEKID1 + EDU: M0 + age at first birth + education. * *p* < 0.1; ** *p* < 0.05; *** *p* < 0.01. (*Source*: SOEP, v37, 1990–2020. Own unweighted estimates)

## Appendix

See Figs. 2 and 3. See Tables

**Table 5:** Sample statistics, time-constant covariates, column %. (*Source*: SOEP, v37, 1990–2020, unweighted estimates)

	Men	Women
*Social class in the year of the first birth*		
Upper service	19	10
Lower service	19	26
Skilled	36	24
Semi-/unskilled	19	23
Not employed	7	18
*Partner’s social class at first birth*		
Upper service	8	13
Lower service	20	12
Skilled	19	22
Semi-/unskilled	16	11
Not employed	13	4
No partner/missing	25	38
*Migration background*		
Native	70	71
Migration background	30	29
*Age at first birth* (mean)	30.8	28.1
*Sample size*		
Persons	2282	2819
Second births	1161	1371

^5^,

**Table 6:** Sample statistics, time-varying covariates by person-months, column %. (Source: SOEP, v37, 1990–2020, unweighted estimates)

	Men	Women
*Mobility*		
Stable	67	38
Upward	9	5
Downward	7	5
Not employed	14	52
Other	2	1
*Education*		
Low	11	12
Medium	63	62
High	24	23
Missing	2	3
*Financial worries*		
No worries	20	22
Stable	53	56
Great worries	27	21
*Region*		
West Germany	78	77
East Germany	22	23
*Sample size*		
Person-months	108,241	132,384
Second births	1161	1371

There are some few (< 1%) missings for financial worries

6,

**Table 7:** Social class of woman by partner’s social class, column %. (*Source*: SOEP, v37, 1990–2020 unweighted estimates)

Social class woman	Social class man				
	Upper service	Lower service	Skilled	Semi-/unskilled	Not employed
Upper service	48	24	17	7	10
Lower service	26	26	18	13	14
Skilled	15	33	44	46	35
Semi-/unskilled	9	14	18	30	30
Not employed	2	3	3	5	11

Unweighted estimates (*n* = 83,654 person-months, Spearman’s correlation coefficient: 0.34)

7,

**Table 8:** Social class of man by partner’s social class, column %. (*Source*: SOEP, v37, 1990–2020 unweighted estimates)

Social class woman	Social class men				
	Upper service	Lower service	Skilled	Semi-/unskilled	Not employed
Upper service	24	13	4	4	3
Lower service	36	39	25	20	13
Skilled	22	24	31	23	18
Semi-/unskilled	10	15	29	33	25
Not employed	8	9	12	20	40

(*n* = 81,669 person-months; Spearman’s correlation coefficient: 0.34)

8.

## Footnotes

1 Goldthorpe (2007: p. 104) distinguishes the following nine classes: I professional and managers, higher grade; II professional and managers, lower grade; IIIa routine nonmanual employees, higher grade; IIIb routine nonmanual employees, lower grade; IVabc small proprietors, self-employed; V technicians, lower-grade supervisors of manual workers; VI skilled manual workers; VIIa semi- and unskilled manual workers (other than in agriculture); and VIIb agricultural workers.

2 In the Latin American context, scholars have also examined the class and fertility nexus (Castro Torres 2021). However, instead of relying on occupation-based class concepts, they measured social class using a large battery of variables, including electricity and water supply.

3 The COVID-19 crisis may have affected fertility in Germany. However, most interviews are conducted at the beginning of the year. As 2020 is the last year when interviews were conducted, only a few 2020 births are included in the analysis.

4 The parity progression ratio that was calculated based on German vital statistics for women ages 45–49 in the year 2018 was about 68% (Statistisches Bundesamt 2019).

5 Note that identification issues can arise if the follow-up is not sufficiently long to observe the “plateau” in the population survival function (see Bremhorst and Lambert 2016; Lambert and Bremhorst 2019).

6 We have conducted further robustness checks and estimated models that excluded age at first childbearing and education (see Fig. 3 in the appendix). The class gradient is less pronounced for the male sample when age at first birth is removed from the model. A model that does not control for education leads to a less pronounced social class gradient for both genders. Note that it is currently debated whether education should be included into models that control for social class (see, for example, Grätz 2022).

7 For illustrative purposes, we have also estimated a median duration time to second birth by social class from Model M1 and W1 (Table 2). Such an approach requires assumptions on the covariate constellations. We estimated values for the following constellation: A German citizen in West Germany with medium education who had the first child between ages 24 and 28, and who did not experience upward mobility. For the male model (M1), the estimated median duration to second birth for the upper service class is 3 years, while it is roughly 4.5 years for the semi-/unskilled class. For the female model (W1), the estimated median duration to second birth for the upper service class is about 3.2 years, while it is about 5 years for the semi-/unskilled class.
